# Crystal structure of 1,2-bis­(2,6-di­methyl­phen­yl)-3-phenyl­guanidine

**DOI:** 10.1107/S2056989015011822

**Published:** 2015-06-27

**Authors:** Hongfei Han, Zhiqiang Guo, Xuehong Wei

**Affiliations:** a The School of Chemistry and Chemical Engineering, Shanxi University, Taiyuan 030006, People’s Republic of China; b Institute of Applied Chemistry, Shanxi University, Taiyuan, Shanxi 030006, People’s Republic of China

**Keywords:** crystal structure, guanidines, hydrogen bonding, π–π overlap

## Abstract

In the title compound, C_23_H_25_N_3_, the dihedral angles between the planes of the benzene ring and the two substituent di­methyl­phenyl rings are 60.94 (7)° and 88.08 (7)°, and the dihedral angle between the planes of the two di­methyl­phenyl rings is 58.01 (7)°. In the crystal, weak C—H⋯N inter­actions exist between adjacent mol­ecules. One of the di­methyl­phenyl rings has a small amount of π–π overlap with the phenyl ring of an adjacent mol­ecule [centroid-to-centroid distance = 3.9631 (12) Å].

## Related literature   

For similar structures of various related compounds, see: Boeré *et al.* (2000 ▸); Brazeau *et al.* (2012 ▸); Ghosh *et al.* (2008 ▸); Han & Huynh (2009 ▸); Chlupatý & Padělková (2014 ▸); Yildirim *et al.* (2007 ▸); Zhang *et al.* (2009 ▸). For applications of guanidines, see: Berlinck (2002 ▸); Heys *et al.* (2000 ▸); Laeckmann *et al.* (2002 ▸); Kelley *et al.* (2001 ▸); Moroni *et al.* (2001 ▸).
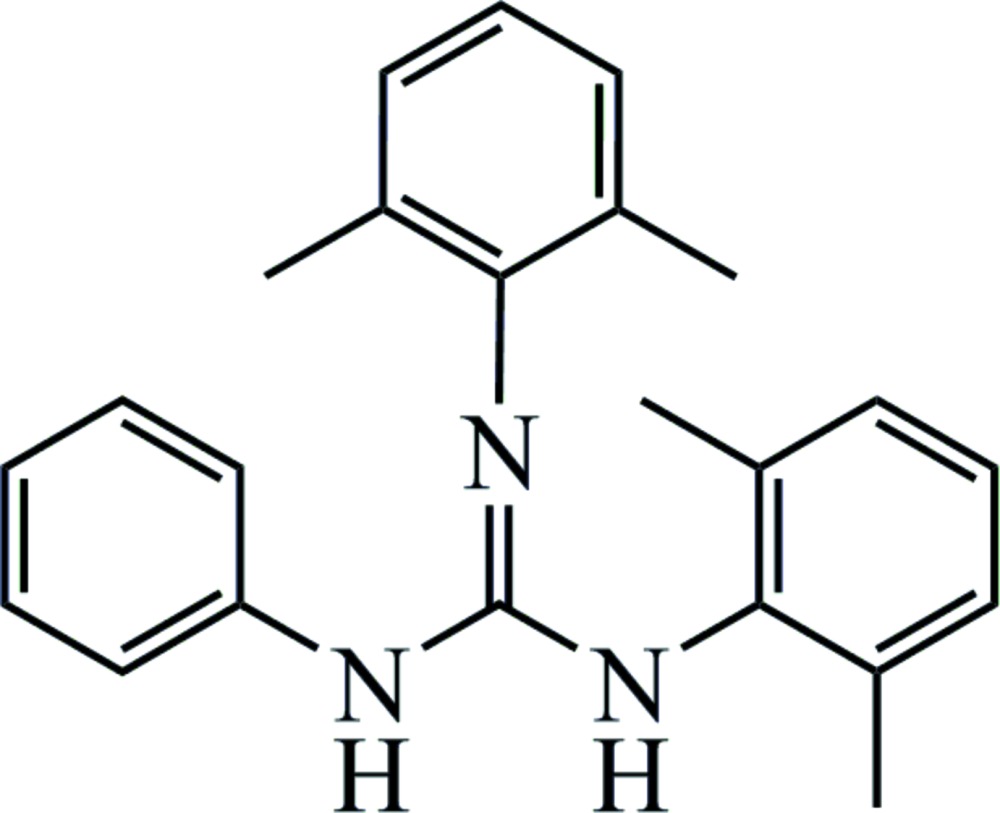


## Experimental   

### Crystal data  

C_23_H_25_N_3_
*M* *_r_* = 343.46
Orthorhombic, 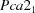

*a* = 19.003 (7) Å
*b* = 7.924 (3) Å
*c* = 13.056 (5) Å
*V* = 1966.0 (13) Å^3^
*Z* = 4
Mo *K*α radiation
μ = 0.07 mm^−1^
*T* = 194 K
0.35 × 0.33 × 0.30 mm

### Data collection  

Bruker SMART CCD area-detector diffractometer
Absorption correction: multi-scan (*SADABS*; Sheldrick, 1996 ▸) *T*_min_ = 0.976, *T*_max_ = 0.980
10737 measured reflections
3547 independent reflections
2649 reflections with *I* > 2σ(*I*)
*R*_int_ = 0.038

### Refinement  

*R*[*F* ^2^ > 2σ(*F* ^2^)] = 0.041
*wR*(*F* ^2^) = 0.104
*S* = 1.02
3547 reflections
239 parameters
1 restraint
H-atom parameters constrained
Δρ_max_ = 0.14 e Å^−3^
Δρ_min_ = −0.14 e Å^−3^

### 

Data collection: *APEX2* (Bruker, 2007 ▸); cell refinement: *SAINT* (Bruker, 2007 ▸); data reduction: *SAINT*; program(s) used to solve structure: *SHELXS97* (Sheldrick, 2008 ▸); program(s) used to refine structure: *SHELXL97* (Sheldrick, 2008 ▸); molecular graphics: *SHELXTL* (Sheldrick, 2008 ▸); software used to prepare material for publication: *publCIF* (Westrip, 2010 ▸).

## Supplementary Material

Crystal structure: contains datablock(s) I. DOI: 10.1107/S2056989015011822/pk2556sup1.cif

Structure factors: contains datablock(s) I. DOI: 10.1107/S2056989015011822/pk2556Isup2.hkl

Supporting information file. DOI: 10.1107/S2056989015011822/pk2556Isup3.cml

**Fig. 1.**
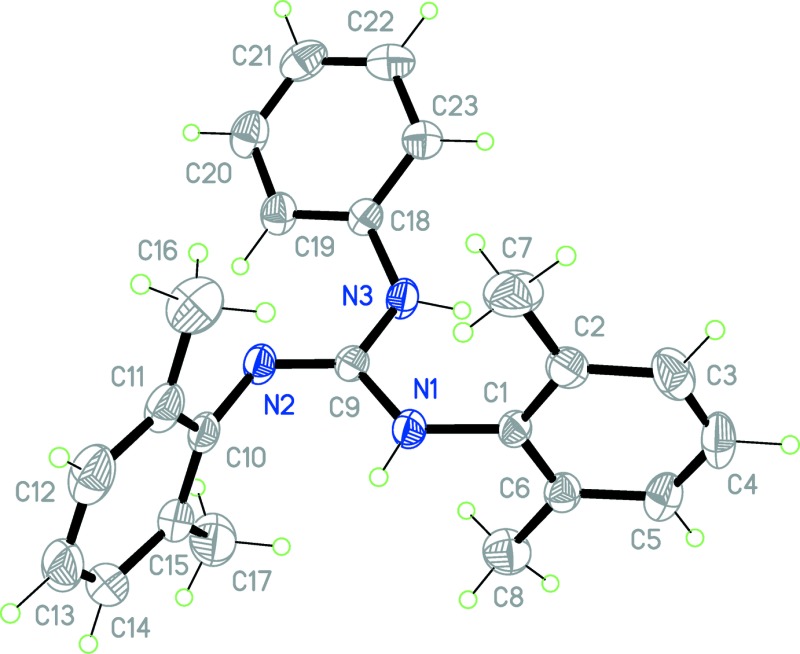
The molecular structure of the title compound. Displacement ellipsoids are drawn at the 50% probability level. H atoms are presented as small spheres of arbitrary radius.

. DOI: 10.1107/S2056989015011822/pk2556fig1.tif
The mol­ecular structure of the title compound. Displacement ellipsoids are drawn at the 50% probability level. H atoms are presented as small spheres of arbitrary radius.

CCDC reference: 1407910

Additional supporting information:  crystallographic information; 3D view; checkCIF report

## Figures and Tables

**Table 1:** Hydrogen-bond geometry (, )

*D*H*A*	*D*H	H*A*	*D* *A*	*D*H*A*
C4H4N2^i^	0.95	2.61	3.457(3)	148

Symmetry code: (i) 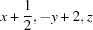.

## supplementary crystallographic information

### S1. Structural commentary

Guanidines are important compounds due to their possible application in medicine, biology and chemistry (Berlinck *et al.*, 2002; Heys *et al.*, 2000). In particular, they have received increasing inter­est as medicinal agents with anti­tumour, anti-hypertensive, anti­glaucoma and cardiotonic activities (Laeckmann *et al.*, 2002; Kelley *et al.*, 2001; Moroni *et al.*, 2001). In search of guanidinato metal complexes and their catalytic behaviors, we synthesized a new substituted guanidine by insertion of phenyl­amine with a symmetric carbodi­imine, the crystal structure of which is presented here. In addition to two examples of phenyl-substituted benzimidazol amines (Ghosh *et al.*, 2008; Yildirim *et al.*, 2007), the title compound is structurally similar to the known compounds, 1-cyclo­hexyl-2,3-di­phenyl­guanidine (Zhang *et al.*, 2009), 1-(2,6-diiso­propyl­phenyl)-2,3-dimesitylguanidine (Brazeau *et al.*, 2012), N,N',N''-tris­(2,6-di­methyl­phenyl)­guanidine (Han & Huynh, 2009), 2-[2,6-Bis(propan-2-yl)phenyl]-1,3-di­cyclo­hexyl­guanidine (Chlupatý & Padělková, 2014) and N,N',N''-tris­(2,6-di-iso­propyl­phenyl)­guanidine (Boere *et al.*, 2000).

The molecular structure of the title compound is illustrated in Fig. 1. The C9—N2 bond in the guanidine unit is 1.266 (2) Å, and is characteristic for a C═N imine double bond. The bond lengths of C9—N1 and C9—N3 are 1.365 (2) and 1.376 (2) Å, showing single bond character (Allen *et al.*, 1987). The N—C9—N angles are 124.15 (18)° (N1—C9—N2), 121.58 (17)° (N2—C9—N3) and 114.26 (17)° (N1—C9—N3), indicating a deviation of the CN_3_ plane from an ideal trigonal planar geometry. The dihedral angles between the planes of the benzene ring and the two substituent di­methyl­phenyl rings are 60.94 (7) and 88.08 (7)°, and the dihedral angle between the planes of the two di­methyl­phenyl rings is 58.01 (7)°. In the crystal, in addition to van der Waals inter­actions, weak C—H···N and N—H···C inter­actions exist between adjacent molecules. One of the di­methyl­phenyl rings has a small amount of π···π overlap with the phenyl ring of an adjacent (1-x, 2-y, 0.5+z) molecule [centroid-to-centroid distance = 3.9631 (12) Å].

### S2. Synthesis and crystallization

To a stirred solution of phenyl­amine (1.863 g, 20 mmol) in hexane was added N,N'-di­methyl­phenyl carbodi­imine (5.007 g, 20 mmol), followed by the addition of the tri­methyl­aluminum (2.5 M, 0.40 mL, 1 mmol). After stirring for 2 h, the white precipitate was collected by suction filtration and recrystallized from hexane-di­ethyl­ether (1:1) solution to obtain colorless crystals of 1,2-bis­(2,6-di­methyl­phenyl)-3-phenyl­guanidine (yield: 90%). Anal. Calc. for C_23_H_25_N_3_: C, 80.43; H, 7.34; N, 12.23. Found: C, 80.32; H, 7.25; N, 12.31%. ^1^H NMR (300 MHz, CDCl_3_, 25 °C) δ p.p.m. 2.35 (*d*, 12H, CH_3_), 5.06 (*s*, 1H, NH), 5.56 (*s*, 1H, NH), 6.91 (*s*, 2H, PhH), 7.00 (*s*, 2H, PhH), 7.14 (*s*, 4H, PhH), 7.28 (*s*, 1H, PhH) , 7.58 (*s*, 2H, PhH).

### S3. Refinement

Crystal data, data collection and structure refinement details are summarized in Table 1. The N—H hydrogen atoms were located in a difference Fourier map and constrained (N—H = 0.87 Å). The C-bound H-atoms were included in calculated positions and treated as riding atoms: C—H = 0.95 - 0.98 Å with *U*_iso_ (H) = 1.2 or 1.5*U*_eq_ (C_Me_).

### Crystal data

**Table:**

C_23_H_25_N_3_	*F*(000) = 736
*M**_r_* = 343.46	*D*_x_ = 1.160 Mg m^−^^3^
Orthorhombic, *P**c**a*2_1_	Mo *K*α radiation, λ = 0.71073 Å
Hall symbol: P 2c -2ac	Cell parameters from 2316 reflections
*a* = 19.003 (7) Å	θ = 2.8–23.5°
*b* = 7.924 (3) Å	µ = 0.07 mm^−^^1^
*c* = 13.056 (5) Å	*T* = 194 K
*V* = 1966.0 (13) Å^3^	Block, colourless
*Z* = 4	0.35 × 0.33 × 0.30 mm

### Data collection

**Table:**

Bruker SMART CCD area-detector diffractometer	3547 independent reflections
Radiation source: fine-focus sealed tube	2649 reflections with *I* > 2σ(*I*)
Graphite monochromator	*R*_int_ = 0.038
φ and ω scans	θ_max_ = 25.5°, θ_min_ = 2.7°
Absorption correction: multi-scan (*SADABS*; Sheldrick, 1996)	*h* = −23→23
*T*_min_ = 0.976, *T*_max_ = 0.980	*k* = −9→5
10737 measured reflections	*l* = −15→15

### Refinement

**Table:**

Refinement on *F*^2^	Primary atom site location: structure-invariant direct methods
Least-squares matrix: full	Secondary atom site location: difference Fourier map
*R*[*F*^2^ > 2σ(*F*^2^)] = 0.041	Hydrogen site location: inferred from neighbouring sites
*wR*(*F*^2^) = 0.104	H-atom parameters constrained
*S* = 1.01	*w* = 1/[σ^2^(*F*_o_^2^) + (0.0564*P*)^2^] where *P* = (*F*_o_^2^ + 2*F*_c_^2^)/3
3547 reflections	(Δ/σ)_max_ = 0.001
239 parameters	Δρ_max_ = 0.14 e Å^−^^3^
1 restraint	Δρ_min_ = −0.14 e Å^−^^3^

### Special details

**Table:**

Geometry. All esds (except the esd in the dihedral angle between two l.s. planes) are estimated using the full covariance matrix. The cell esds are taken into account individually in the estimation of esds in distances, angles and torsion angles; correlations between esds in cell parameters are only used when they are defined by crystal symmetry. An approximate (isotropic) treatment of cell esds is used for estimating esds involving l.s. planes.
Refinement. Refinement of F^2^ against ALL reflections. The weighted R-factor wR and goodness of fit S are based on F^2^, conventional R-factors R are based on F, with F set to zero for negative F^2^. The threshold expression of F^2^ > 2sigma(F^2^) is used only for calculating R-factors(gt) etc. and is not relevant to the choice of reflections for refinement. R-factors based on F^2^ are statistically about twice as large as those based on F, and R- factors based on ALL data will be even larger.

### Fractional atomic coordinates and isotropic or equivalent isotropic displacement parameters (Å^2^)

**Table:**

	*x*	*y*	*z*	*U*_iso_*/*U*_eq_	
N1	0.51390 (8)	0.8726 (2)	0.22933 (14)	0.0499 (4)	
H1	0.5001	0.8129	0.2815	0.060*	
N2	0.39761 (8)	0.9417 (2)	0.19326 (13)	0.0470 (4)	
N3	0.48706 (8)	1.0319 (2)	0.08715 (13)	0.0494 (5)	
H3	0.5300	1.0109	0.0680	0.059*	
C1	0.58760 (9)	0.8820 (3)	0.21187 (15)	0.0414 (5)	
C2	0.62483 (11)	1.0196 (3)	0.24776 (18)	0.0535 (6)	
C3	0.69708 (12)	1.0198 (4)	0.2354 (2)	0.0693 (7)	
H3A	0.7238	1.1136	0.2586	0.083*	
C4	0.73035 (12)	0.8867 (4)	0.1904 (2)	0.0695 (7)	
H4	0.7801	0.8879	0.1840	0.083*	
C5	0.69327 (12)	0.7521 (4)	0.15447 (18)	0.0612 (6)	
H5	0.7173	0.6614	0.1223	0.073*	
C6	0.62094 (10)	0.7464 (3)	0.16442 (17)	0.0482 (5)	
C7	0.58943 (16)	1.1640 (3)	0.3014 (2)	0.0825 (8)	
H7A	0.5631	1.1213	0.3605	0.124*	
H7B	0.5570	1.2202	0.2539	0.124*	
H7C	0.6250	1.2448	0.3247	0.124*	
C8	0.57928 (15)	0.5980 (3)	0.1261 (2)	0.0732 (7)	
H8A	0.5423	0.6375	0.0796	0.110*	
H8B	0.5578	0.5392	0.1843	0.110*	
H8C	0.6106	0.5204	0.0894	0.110*	
C9	0.46259 (9)	0.9480 (3)	0.17228 (15)	0.0392 (4)	
C10	0.37336 (10)	0.8594 (3)	0.28195 (16)	0.0470 (5)	
C11	0.36221 (11)	0.9503 (4)	0.37126 (18)	0.0592 (7)	
C12	0.32944 (14)	0.8739 (5)	0.4525 (2)	0.0830 (9)	
H12	0.3214	0.9366	0.5133	0.100*	
C13	0.30812 (15)	0.7093 (6)	0.4476 (3)	0.0950 (12)	
H13	0.2852	0.6584	0.5044	0.114*	
C14	0.32015 (14)	0.6189 (4)	0.3606 (3)	0.0918 (11)	
H14	0.3059	0.5041	0.3580	0.110*	
C15	0.35250 (11)	0.6897 (3)	0.2757 (2)	0.0628 (7)	
C16	0.38379 (16)	1.1319 (4)	0.3781 (2)	0.0814 (9)	
H16A	0.3731	1.1888	0.3133	0.122*	
H16B	0.4344	1.1389	0.3916	0.122*	
H16C	0.3580	1.1868	0.4338	0.122*	
C17	0.36204 (16)	0.5931 (4)	0.1775 (3)	0.0942 (10)	
H17A	0.3381	0.6526	0.1217	0.141*	
H17B	0.3419	0.4799	0.1850	0.141*	
H17C	0.4123	0.5839	0.1619	0.141*	
C18	0.44829 (10)	1.1495 (3)	0.02788 (14)	0.0437 (5)	
C19	0.37790 (11)	1.1326 (3)	0.00775 (16)	0.0546 (6)	
H19	0.3529	1.0372	0.0326	0.065*	
C20	0.34337 (13)	1.2550 (3)	−0.04892 (17)	0.0641 (7)	
H20	0.2943	1.2446	−0.0611	0.077*	
C21	0.37888 (15)	1.3896 (3)	−0.0873 (2)	0.0685 (7)	
H21	0.3546	1.4731	−0.1258	0.082*	
C22	0.44980 (15)	1.4048 (3)	−0.07045 (19)	0.0666 (7)	
H22	0.4749	1.4976	−0.0985	0.080*	
C23	0.48466 (11)	1.2853 (3)	−0.01269 (16)	0.0533 (5)	
H23	0.5338	1.2964	−0.0007	0.064*	

### Atomic displacement parameters (Å^2^)

**Table:**

	*U*^11^	*U*^22^	*U*^33^	*U*^12^	*U*^13^	*U*^23^
N1	0.0348 (8)	0.0591 (10)	0.0557 (10)	0.0034 (8)	0.0009 (8)	0.0171 (9)
N2	0.0339 (9)	0.0611 (11)	0.0460 (10)	0.0061 (8)	0.0012 (7)	0.0097 (9)
N3	0.0358 (9)	0.0664 (12)	0.0461 (9)	0.0083 (8)	0.0054 (7)	0.0140 (10)
C1	0.0336 (10)	0.0483 (11)	0.0424 (11)	−0.0002 (9)	−0.0060 (8)	0.0072 (10)
C2	0.0533 (12)	0.0528 (13)	0.0543 (13)	−0.0053 (11)	−0.0068 (11)	0.0059 (11)
C3	0.0564 (14)	0.0834 (19)	0.0682 (15)	−0.0248 (14)	−0.0165 (13)	0.0080 (15)
C4	0.0356 (11)	0.111 (2)	0.0622 (15)	−0.0066 (13)	−0.0028 (11)	0.0113 (17)
C5	0.0477 (13)	0.0820 (18)	0.0540 (13)	0.0115 (12)	0.0023 (10)	−0.0007 (13)
C6	0.0432 (11)	0.0551 (13)	0.0461 (11)	0.0001 (10)	−0.0039 (10)	0.0016 (11)
C7	0.0967 (19)	0.0517 (14)	0.099 (2)	0.0000 (14)	−0.0121 (17)	−0.0118 (17)
C8	0.0800 (18)	0.0600 (17)	0.0795 (17)	−0.0056 (14)	−0.0001 (14)	−0.0135 (14)
C9	0.0368 (10)	0.0428 (11)	0.0379 (10)	0.0029 (9)	−0.0032 (9)	−0.0003 (9)
C10	0.0295 (9)	0.0615 (14)	0.0498 (12)	0.0091 (10)	0.0007 (9)	0.0086 (11)
C11	0.0449 (13)	0.0842 (18)	0.0483 (13)	0.0202 (13)	0.0008 (10)	0.0096 (13)
C12	0.0684 (18)	0.127 (3)	0.0534 (15)	0.0326 (18)	0.0107 (13)	0.0181 (18)
C13	0.0656 (17)	0.129 (3)	0.091 (2)	0.0279 (19)	0.0308 (16)	0.058 (2)
C14	0.0606 (16)	0.078 (2)	0.137 (3)	0.0074 (15)	0.0213 (18)	0.044 (2)
C15	0.0420 (12)	0.0628 (15)	0.0837 (17)	0.0046 (11)	0.0078 (12)	0.0096 (15)
C16	0.088 (2)	0.085 (2)	0.0710 (17)	0.0123 (17)	−0.0064 (15)	−0.0176 (17)
C17	0.0784 (18)	0.0710 (18)	0.133 (3)	−0.0084 (15)	0.015 (2)	−0.023 (2)
C18	0.0450 (11)	0.0506 (13)	0.0355 (10)	0.0058 (9)	0.0019 (9)	0.0008 (10)
C19	0.0453 (12)	0.0729 (17)	0.0455 (12)	0.0040 (11)	−0.0026 (9)	0.0083 (12)
C20	0.0533 (13)	0.0902 (19)	0.0487 (12)	0.0137 (13)	−0.0040 (11)	0.0067 (14)
C21	0.0819 (18)	0.0696 (18)	0.0541 (14)	0.0251 (15)	−0.0055 (13)	0.0062 (14)
C22	0.090 (2)	0.0497 (15)	0.0606 (15)	0.0046 (13)	−0.0032 (13)	0.0051 (13)
C23	0.0574 (13)	0.0517 (13)	0.0509 (12)	−0.0007 (11)	−0.0028 (11)	−0.0014 (12)

### Geometric parameters (Å, º)

**Table:**

N1—C9	1.365 (2)	C11—C12	1.370 (4)
N1—C1	1.421 (2)	C11—C16	1.499 (4)
N1—H1	0.8700	C12—C13	1.368 (5)
N2—C9	1.266 (2)	C12—H12	0.9500
N2—C10	1.407 (3)	C13—C14	1.363 (5)
N3—C9	1.376 (2)	C13—H13	0.9500
N3—C18	1.418 (3)	C14—C15	1.386 (4)
N3—H3	0.8699	C14—H14	0.9500
C1—C2	1.381 (3)	C15—C17	1.503 (4)
C1—C6	1.393 (3)	C16—H16A	0.9800
C2—C3	1.382 (3)	C16—H16B	0.9800
C2—C7	1.501 (4)	C16—H16C	0.9800
C3—C4	1.363 (4)	C17—H17A	0.9800
C3—H3A	0.9500	C17—H17B	0.9800
C4—C5	1.362 (3)	C17—H17C	0.9800
C4—H4	0.9500	C18—C19	1.370 (3)
C5—C6	1.381 (3)	C18—C23	1.385 (3)
C5—H5	0.9500	C19—C20	1.385 (3)
C6—C8	1.503 (3)	C19—H19	0.9500
C7—H7A	0.9800	C20—C21	1.358 (3)
C7—H7B	0.9800	C20—H20	0.9500
C7—H7C	0.9800	C21—C22	1.371 (4)
C8—H8A	0.9800	C21—H21	0.9500
C8—H8B	0.9800	C22—C23	1.380 (3)
C8—H8C	0.9800	C22—H22	0.9500
C10—C11	1.387 (3)	C23—H23	0.9500
C10—C15	1.405 (3)		
			
C9—N1—C1	126.42 (17)	C12—C11—C16	120.2 (3)
C9—N1—H1	116.8	C10—C11—C16	120.5 (2)
C1—N1—H1	116.8	C13—C12—C11	121.3 (3)
C9—N2—C10	121.07 (16)	C13—C12—H12	119.3
C9—N3—C18	125.64 (16)	C11—C12—H12	119.3
C9—N3—H3	117.2	C14—C13—C12	119.3 (3)
C18—N3—H3	117.2	C14—C13—H13	120.3
C2—C1—C6	121.78 (17)	C12—C13—H13	120.3
C2—C1—N1	119.46 (19)	C13—C14—C15	121.9 (3)
C6—C1—N1	118.63 (17)	C13—C14—H14	119.0
C1—C2—C3	118.0 (2)	C15—C14—H14	119.0
C1—C2—C7	122.0 (2)	C14—C15—C10	117.8 (3)
C3—C2—C7	119.9 (2)	C14—C15—C17	122.0 (3)
C4—C3—C2	120.7 (2)	C10—C15—C17	120.2 (2)
C4—C3—H3A	119.7	C11—C16—H16A	109.5
C2—C3—H3A	119.7	C11—C16—H16B	109.5
C5—C4—C3	121.0 (2)	H16A—C16—H16B	109.5
C5—C4—H4	119.5	C11—C16—H16C	109.5
C3—C4—H4	119.5	H16A—C16—H16C	109.5
C4—C5—C6	120.5 (2)	H16B—C16—H16C	109.5
C4—C5—H5	119.7	C15—C17—H17A	109.5
C6—C5—H5	119.7	C15—C17—H17B	109.5
C5—C6—C1	118.0 (2)	H17A—C17—H17B	109.5
C5—C6—C8	121.2 (2)	C15—C17—H17C	109.5
C1—C6—C8	120.80 (18)	H17A—C17—H17C	109.5
C2—C7—H7A	109.5	H17B—C17—H17C	109.5
C2—C7—H7B	109.5	C19—C18—C23	119.3 (2)
H7A—C7—H7B	109.5	C19—C18—N3	123.2 (2)
C2—C7—H7C	109.5	C23—C18—N3	117.43 (18)
H7A—C7—H7C	109.5	C18—C19—C20	119.8 (2)
H7B—C7—H7C	109.5	C18—C19—H19	120.1
C6—C8—H8A	109.5	C20—C19—H19	120.1
C6—C8—H8B	109.5	C21—C20—C19	120.8 (2)
H8A—C8—H8B	109.5	C21—C20—H20	119.6
C6—C8—H8C	109.5	C19—C20—H20	119.6
H8A—C8—H8C	109.5	C20—C21—C22	119.9 (2)
H8B—C8—H8C	109.5	C20—C21—H21	120.1
N2—C9—N1	124.15 (18)	C22—C21—H21	120.1
N2—C9—N3	121.58 (17)	C21—C22—C23	120.0 (2)
N1—C9—N3	114.26 (16)	C21—C22—H22	120.0
C11—C10—C15	120.2 (2)	C23—C22—H22	120.0
C11—C10—N2	120.1 (2)	C22—C23—C18	120.2 (2)
C15—C10—N2	119.2 (2)	C22—C23—H23	119.9
C12—C11—C10	119.4 (3)	C18—C23—H23	119.9
			
C9—N1—C1—C2	82.9 (3)	C15—C10—C11—C12	−1.3 (3)
C9—N1—C1—C6	−101.1 (2)	N2—C10—C11—C12	170.91 (19)
C6—C1—C2—C3	0.0 (3)	C15—C10—C11—C16	−179.4 (2)
N1—C1—C2—C3	175.9 (2)	N2—C10—C11—C16	−7.3 (3)
C6—C1—C2—C7	−178.4 (2)	C10—C11—C12—C13	0.7 (4)
N1—C1—C2—C7	−2.5 (3)	C16—C11—C12—C13	178.9 (3)
C1—C2—C3—C4	−0.9 (4)	C11—C12—C13—C14	0.5 (4)
C7—C2—C3—C4	177.5 (2)	C12—C13—C14—C15	−1.0 (5)
C2—C3—C4—C5	1.4 (4)	C13—C14—C15—C10	0.4 (4)
C3—C4—C5—C6	−1.0 (4)	C13—C14—C15—C17	−176.4 (3)
C4—C5—C6—C1	0.1 (3)	C11—C10—C15—C14	0.7 (3)
C4—C5—C6—C8	−179.4 (2)	N2—C10—C15—C14	−171.5 (2)
C2—C1—C6—C5	0.4 (3)	C11—C10—C15—C17	177.6 (2)
N1—C1—C6—C5	−175.5 (2)	N2—C10—C15—C17	5.4 (3)
C2—C1—C6—C8	179.9 (2)	C9—N3—C18—C19	−37.2 (3)
N1—C1—C6—C8	4.0 (3)	C9—N3—C18—C23	144.1 (2)
C10—N2—C9—N1	2.6 (3)	C23—C18—C19—C20	−2.8 (3)
C10—N2—C9—N3	−178.32 (19)	N3—C18—C19—C20	178.5 (2)
C1—N1—C9—N2	−177.0 (2)	C18—C19—C20—C21	1.8 (3)
C1—N1—C9—N3	3.9 (3)	C19—C20—C21—C22	0.3 (4)
C18—N3—C9—N2	15.3 (3)	C20—C21—C22—C23	−1.4 (4)
C18—N3—C9—N1	−165.6 (2)	C21—C22—C23—C18	0.4 (4)
C9—N2—C10—C11	94.0 (2)	C19—C18—C23—C22	1.8 (3)
C9—N2—C10—C15	−93.7 (2)	N3—C18—C23—C22	−179.54 (19)

### Hydrogen-bond geometry (Å, º)

**Table:**

*D*—H···*A*	*D*—H	H···*A*	*D*···*A*	*D*—H···*A*
C4—H4···N2^i^	0.95	2.61	3.457 (3)	148

Symmetry code: (i) *x*+1/2, −*y*+2, *z*.
