# Supplementary figures and images for: Crystal structure of 1,2-bis­(2,6-di­methyl­phen­yl)-3-phenyl­guanidine
Source: Acta Crystallogr E Crystallogr Commun. 2015 Jun 27;71(Pt 7):o506–7. doi: 10.1107/S2056989015011822 (PMC4518950; doi:10.1107/S2056989015011822)

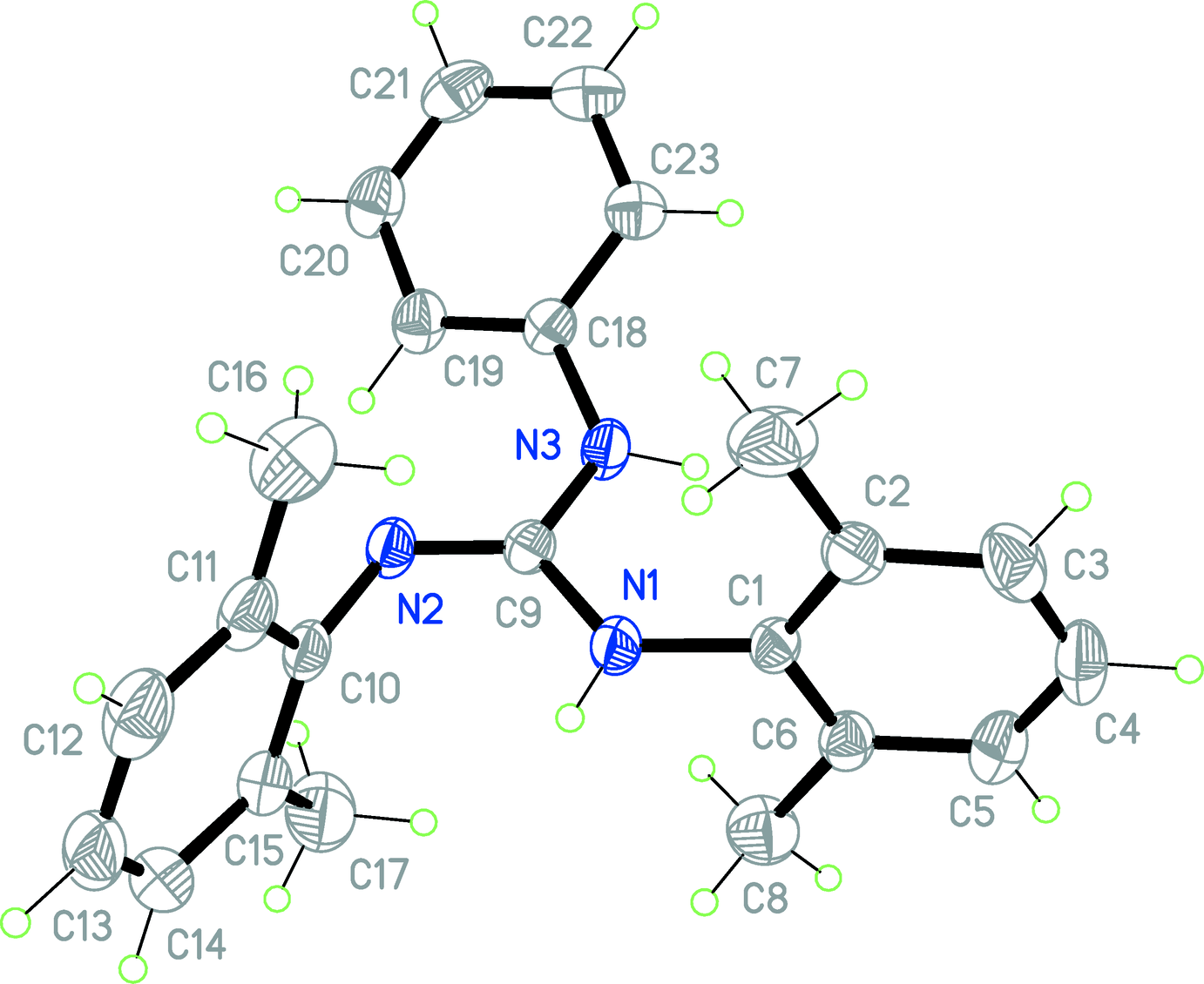

Supplement: Supplementary file 4 [file e-71-0o506-fig1.tif]
